# Sequence Ontology Annotation Guide

**DOI:** 10.1002/cfg.446

**Published:** 2004-12

**Authors:** Karen Eilbeck, Suzanna E. Lewis

**Affiliations:** Department of Molecular and Cellular Biology Life Sciences Addition University of California Berkeley California 94729-3200 USA

## Abstract

This Sequence Ontology (SO) [13] aims to unify the way in which we describe sequence annotations, by providing a controlled vocabulary of terms and the relationships between them. Using SO terms to label the parts of sequence annotations greatly facilitates downstream analyses of their contents, as it ensures that annotations produced by different groups conform to a single standard. This greatly facilitates analyses of annotation contents and characteristics, e.g. comparisons of UTRs, alternative splicing, etc. Because SO also specifies the relationships between features, e.g. *part_of, kind_of*, annotations described with SO terms are also better substrates for validation and visualization software.

This document provides a step-by-step guide to producing a SO compliant file describing a sequence annotation. We illustrate this by using an annotated gene as an example. First we show where the terms needed to describe the gene's features are located in SO and their relationships to one another. We then show line by line how to format the file to construct a SO compliant annotation of this gene.


**Comparative and Functional Genomics**
*Comp Funct Genom* 2004; **5**: 642–647.
Published online in Wiley InterScience (www.interscience.wiley.com). **DOI:** 10.1002/cfg.446

Conference Paper

### Sequence Ontology Annotation Guide

Karen Eilbeck* and Suzanna E. Lewis
*Department of Molecular and Cellular Biology, Life Sciences Addition, University of California, Berkeley, CA 94729-3200, USA*

**Correspondence to:*
*Karen Eilbeck, Department of Molecular and Cellular Biology, Life Sciences Addition, University of California, Berkeley, California, 94729-3200, USA.*
*E-mail: eilbeck@fruitfly.org*

### Abstract

**This Sequence Ontology (SO) [13] aims to unify the way in which we describe sequence annotations, by providing a controlled vocabulary of terms and the relationships between them. Using SO terms to label the parts of sequence annotations greatly facilitates downstream analyses of their contents, as it ensures that annotations produced by different groups conform to a single standard. This greatly facilitates analyses of annotation contents and characteristics, e.g. comparisons of UTRs, alternative splicing, etc. Because SO also specifies the relationships between features, e.g. *part_of*, *kind_of*, annotations described with SO terms are also better substrates for validation and visualization software.**

**This document provides a step-by-step guide to producing a SO compliant file describing a sequence annotation. We illustrate this by using an annotated gene as an example. First we show where the terms needed to describe the gene's features are located in SO and their relationships to one another. We then show line by line how to format the file to construct a SO compliant annotation of this gene. Copyright © 2005 John Wiley & Sons, Ltd.**

Received: 17 November 2004
Revised: 24 November 2004
Accepted: 25 November 2004

**Keywords:** ontology; annotation; genomics

### What is sequence annotation?

Sequence annotations provide explanatory notes and critical commentary about a sequence, e.g. indicating where transcription occurs and where a regulatory region lies. They may arise from bioinformatics analysis, wet-bench analysis, or a combination of both by an expert biologist. Genomic DNA sequence is most commonly associated with annotation, but any biological sequence may be annotated, e.g. a microarray probe or an mRNA sequence. These annotations allow us to connect what we know about the biology, and results of experiments, with the actual sequence. It enables us to readily locate features on the sequence and relate them to other features. We can also assign additional properties to these features, e.g. where (in which cell types or tissues) a gene is expressed.

There has been a proliferation of exchange formats to reflect the varying needs of the community over time. The three large genome databanks, DDBJ [10], EMBL [8] and GenBank [1] distribute their sequences as flat files, and all use an agreed-upon feature table [3] to name the features of their annotations. As model organism groups needed to exchange complex data models, other formats appeared, such as game-XML [4] and GFF [6,7]. Rendering of genomic annotation into graphical views also became an important issue, and formats such as Bioinformatics Sequence Markup Language (BSML) [2] also appeared. All of these exchange formats rely upon simple controlled lists of key words that enumerate permissible feature types.

Currently there is no single, standardized means for describing an annotation. This makes annotation exchange and analysis a much more complicated task than it need be. More importantly, what distinguishes SO from the keyword lists (feature tables) used by the big genome databases is that it formally specifies the sub-class, membership (mereological), and topological relationships that exist between the

Copyright © 2005 John Wiley & Sons, Ltd.

terms. Specifying these relationships in a principled way provides the basis for a readily extensible object-oriented data model. Software need not be aware of the terms themselves, but need only be aware of the nature of the possible relationships between them. This completely inverts the previous paradigm where the relationships were essentially hard-coded or implicit in their physical placement in the file. Thus, using SO, both data exchange formats and the software that manipulates their contents need only be 'aware' of the underlying relatedness of the features. Moreover, the variety of possible relationship types is much more constrained and their behavior is formally specified, making it possible to readily include additional terms or move terms about in the ontology without re-writing any code for parsing and rendering.

There are several tools for genome browsing, annotation, curation [9,12,14], and viewing a gene via a genome browser graphically demonstrates the relationships between the features. Figure 1 depicts the *Drosophila melanogaster* gene CG10188. This gene is located on the reverse strand and has two annotated transcripts and a total of four exons, three of which are coding (opaque) and one is non-coding (transparent). If an exon includes any coding sequence at all, even one base, it is categorized as coding. There is also a transposable element of type Cr1a located within the intron of the transcripts.

### The Sequence Ontology

In SO each term is defined with a descriptive definition, agreed upon by the community, and the relationships the term has to other terms provide a logical description. Describing the relationship between terms in this way restricts how they can be applied to describe a sequence. For example, the ontology states that an *intron* is **part_of** a *primary_transcript*, and a *primary_transcript* **is_a** *transcript*, whereas an *mRNA* **is_a** *processed_transcript*, and a *processed_transcript* **is_a** *transcript*. So it would be illogical to state that an *intron* is **part_of** an *mRNA*. This is illustrated by following the relationships between the terms in Figure 2.

Properties are concepts that are not locatable on the sequence, but describe an aspect of a feature located on the sequence. For example, SO has terms to describe attributes of a feature, such as the kind of regulation a *gene* undergoes, which include *maternally_imprinted* and *negatively_autoregulated*. Additionally, there are terms to describe chromosome variation and consequences of mutation.

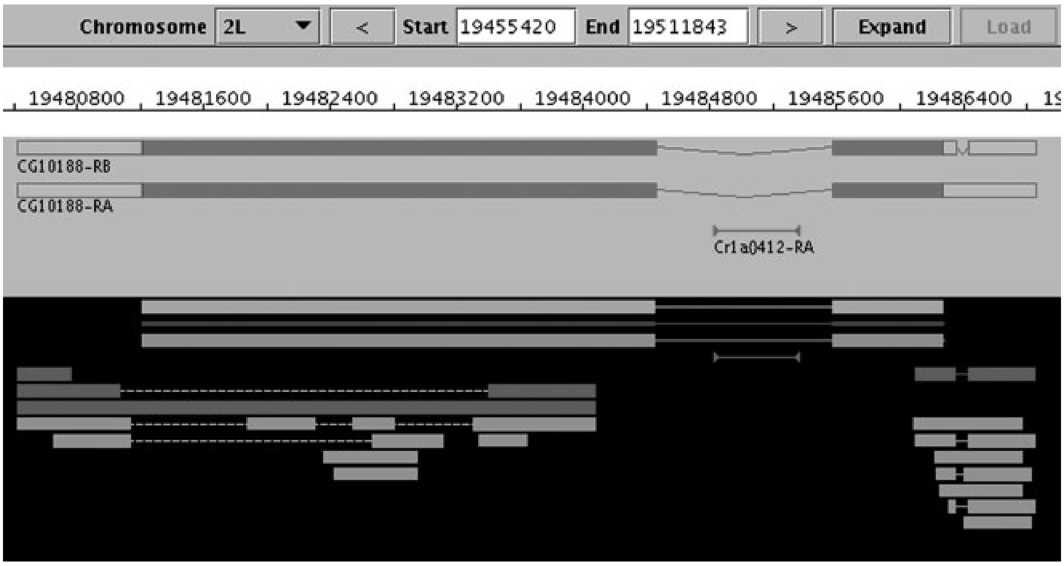

**Figure 1.** Representation of the gene CG10188 of the *Drosophila melanogaster* genome, using the Apollo genome browser [9]. This figure shows both the annotations, in the top tier, and various computational evidence, in the lower tier. The gene is on the reverse (bottom) strand of the DNA sequence

Copyright © 2005 John Wiley & Sons, Ltd. 

### Representing SO instances

The ontology describes our current knowledge of biological sequences and their relationships to one another (since every feature is itself a sequence). But it does not describe actual specific instances of sequence, for this a separate framework; i.e. a flat-file format or database schema is required. As mentioned earlier, there are many formats and representations that are suitable for this. For example, SO terms may be used to type specific features (i.e. sequences) in a relational database or to label the features in a flat file format such as GFF3, or in a hierarchical markup such as XML.

There are currently two data exchange formats that rely on SO to type their features: Generic Feature Format 3 (GFF3 [7]) and the Chado relational schema from the Generic Model Organism Database group (GMOD [5]). In the remainder of this document, GFF3 is used to illustrate how biological features are modelled using SO. The format of GFF3 is outlined in Table 1.

The GFF3 file denoted in Figure 3 represents the gene CG10188 shown graphically in Figure 1. The file starts with three comment lines that state the version of the format and also the region of the genome being annotated, in this case a span of chromosome 2L. Comment lines begin with '# #'. For clarity, the title of each column of the file format is also included in a comment line.

The first line of the actual annotation describes the *gene* that is being annotated. In this case the landmark is the chromosome arm 2L. The source field is undefined and the type of feature is *gene*. Although it is not always clear where a gene begins and ends, in this annotation, it is the five-prime-most base of the transcripts to the three-prime-most base. The score is not defined; the gene is located on the reverse strand; and this feature does not have a phase. The attributes recorded for this gene show its unique identifier, its name and a property. The second line of the annotation describes the first of the transcripts (CG10188-RA) of the gene. This *transcript* is typed as *mRNA*, as this term gives more information to the user than *transcript*. Because of the laws of transitivity, *mRNA* inherits the relationships and attributes of *transcript* via *processed_transcript*. This can be demonstrated by tracing the **is_a** relationships from *mRNA* to *transcript* in Figure 2. The Parent tag has

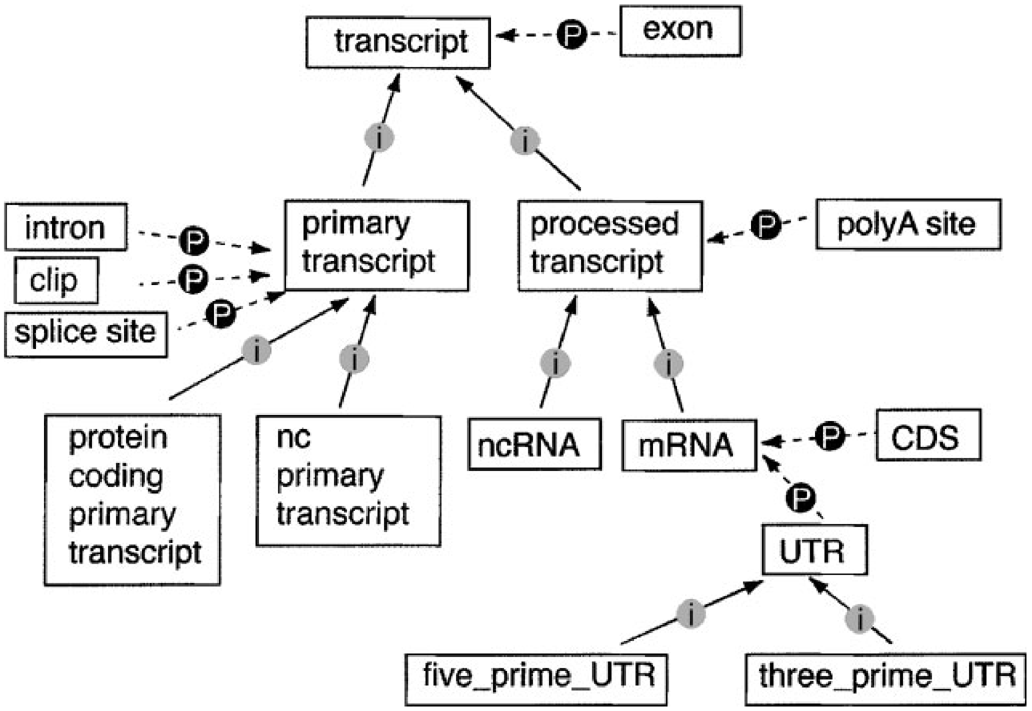

**Figure 2.** A selection of the terms in SO that relates kinds of transcripts, and their parts. The relationships shown are **is_a** ('i'), which provides the sub-type hierarchy, and **part_of** ('P'), which produces meronomies

Copyright © 2005 John Wiley & Sons, Ltd. 

**Table 1.** A description of the columns of the GFF3 format. The format consists of one line per sequence feature with nine columns per line. If a column is not defined, the '.' symbol is used. Comment lines begin with '##'

| | | |
|---|---|---|
| 1 | *seqid* | The landmark to which the coordinates are given. |
| 2 | *source* | The procedure that produced the feature. For example, the name of a piece of software or another database may be appropriate. Not all features have a source. |
| 3 | *type* | The type of feature using either a term name or accession number from the Sequence Ontology. |
| 4 | *begin* | The *begin* coordinate of the feature relative to the landmark given in column 2 where 1-based integer coordinates are used. |
| 5 | *end* | The *end* coordinate of the feature relative to the landmark given in column 1 where 1-based integer coordinates are used. |
| 6 | *score* | The score attributed to the feature if required. |
| 7 | *strand* | The direction of the annotation. |
| 8 | *phase* | The phase of the feature. Not all features have a phase. |
| 9 | *attributes* | The attributes of the feature are recorded as tag-value pairs and multiple attributes are separated by semi-colons. Lower case tags are unrestricted, but upper case tags are reserved for special meanings. There are several tags with predefined meanings: ID is the identifier for the feature and the value of this tag must be unique within the document. Name is the tag used for display purposes for the feature so it does not have to be unique. Another commonly used reserved tag is Parent, which is used to capture **part_of** relations. The value of this tag is the ID of the 'parent'. |

been used to show that the *mRNA* feature is **part_of** the *gene*.

The next five lines in the annotation identify the protein coding and non-protein coding portions of the *mRNA*. This is done by defining the *five_prime_UTR*, the *CDS* and the three*_prime_UTR*. The *CDS* is defined as 'a contiguous sequence which begins with and includes a start codon and ends with and includes a stop codon'. The *CDS* sequence in the annotation must therefore contain the start and stop codons. As can be seen in Figure 1, the *CDS* and the *five_prime-UTR* of the transcript CD10188 span more than one exon. When using these terms, it is therefore necessary to use split locations, which reflect the exon boundaries. Thus, the *five_prime_UTR* takes up two lines in the file but has a single ID. The five_prime_UTR is a **part_of** the *mRNA*, shown by the Parent tag-value pair. The following two lines show the coding portion of two exons, and are typed with the term *CDS*. This is also a split location annotation, and the CDS is part_of the mRNA. The final portion of the last exon is the *three_prime_UTR*, which is the sequence following the stop codon to the end of the transcript.

There is often more than one way to annotate sequence using SO. The second transcript, CG10188-RB, is annotated with *exons* to demonstrate this point. An *exon* is a **part_of** a *transcript*, so by inheritance it is also part of an *mRNA*. The *mRNA* has two *exons*, which, unlike the *CDS* annotations, have unique IDs. To be able to differentiate between the coding and non-coding portions of the transcript, more information is needed. The two lines following the exons label the locations of the UTR.

Both ways of annotating these transcripts would validate, as they are both true to the relationships in the ontology. However, it is common practice in the model organism community to use the first method and annotate to the *CDS* rather than the *exons*, as the non-coding exon structure is often unknown.

Although the introns are not explicitly annotated in this example, as they are implicit, it is possible to include them. These examples have focused on the parts of transcripts and genes, but all features that can be located on the sequence may be annotated in this way. There is a transposable element located in an intron of this gene. Transposable elements are not strictly parts of genes, although they may be located among them. The final row of Figure 3 shows the annotation of a *transposable_element*.

Relationships are recorded in the final column of the GFF3 file. Currently, only the **part_of** relationship is strictly enforced in GFF3. Other relationships may be created. For deeper annotation that details attributes of the features, the tag-value pairs are appropriate for attaching these properties to the feature. The property relation is the tag, and the term is the value, e.g. a transcript may be negatively autoregulated, so the tag-value pair would be

Copyright © 2005 John Wiley & Sons, Ltd.

##GFF-version3

##sequence region 2L 19486843–19480420

| ##seqid | source | type | begin | end | score | strand | phase | attribute |
|---|---|---|---|---|---|---|---|---|
| 2L | . | gene | 19480420 | 19486843 | . | – | . | ID=0001; Name=CG10188; has_genome_location =nuclear_gene; |
| 2L | . | mRNA | 19480420 | 19486843 | . | – | . | ID=0002; Name=CG10188-RA; Parent=0001; |
| 2L | . | five_prime_UTR | 19486435 | 19486843 | . | – | . | ID=0003; Parent=0002; |
| 2L | . | five_prime_UTR | 19486270 | 19486348 | . | – | . | ID=0003; Parent=0002; |
| 2L | . | CDS | 19485573 | 19486269 | . | – | . | ID=0004; Name=CG10188-cdsA; Parent=0002; |
| 2L | . | CDS | 19481212 | 19484444 | . | – | . | ID=0004; Name=CG10188-cdsA; Parent=0002; |
| 2L | . | three_prime_UTR | 19480420 | 19481213 | . | – | . | ID=0005; Parent=0002; |
| 2L | . | mRNA | 19480420 | 19486843 | . | – | . | ID=0006; Name=CG10188-RB; Parent=0001; |
| 2L | . | exon | 19485573 | 19486843 | . | – | . | ID=0007; Name=CG10188-1; Parent=0006; |
| 2L | . | exon | 19480420 | 19484444 | . | – | . | ID=0008; Name=CG10188-2; Parent=0006; |
| 2L | . | five_prime_UTR | 19486270 | 19486843 | . | – | . | ID=0009; Parent=0006; |
| 2L | . | three_prime_UTR | 19480420 | 19481213 | . | – | . | ID=0005; Parent=0006; |
| 2L | . | transposable_element | 19484822 | 19485356 | . | – | . | ID=0010; Name=Cr1a{}412-RA; |

**Figure 3.** The features of the gene CG10188 represented as GFF3. The first transcript (CG10188-RA) is annotated as a split CDS feature and respective UTR, whereas the second transcript (CG10188-RB) is annotated as exons, and the coding portion implied using the UTR. The transposable element Cr1a{}412-RA is also annotated in this region but is not related to the gene by a **part_of** relationship

Copyright © 2005 John Wiley & Sons, Ltd. 

is_regulated = negatively_autoregulated. The gene feature in Table 1 is annotated with a tag-value pair corresponding to a property relation and ontology term (has_genome_location=nuclear_gene).

### Validating SO annotations

The flexibility of the SO allows researchers to annotate their sequence in many different ways and still be consistent with the ontology. Validation is a process whereby the content of the annotation is compared to the knowledge that is captured in the ontology. Each assertion that is made in the annotation must be found in the ontology for the annotation to validate. An annotation that had introns as parts of the mRNA, for example, would be in violation of the SO model.

### Contributing to SO

SO develops and improves through use and feedback from the community. A mailing list exists where people using SO can exchange ideas and comments. Members can join at the SO website (**http://song.sourceforge.net**), and non-members may post to **song-devel@lists.sourceforge.net**. SO is a member of the Open Biological Ontologies (OBO) [11].

### References

1. Benson DA, Karsch-Mizrachi I, Lipman DJ, J, Wheeler DL. 2003. GenBank. *Nucleic Acids Res* **31**: 23–27.
2. BSML documentation; **www.bsml.org**.
3. DDBJ/EMBL/GenBank Feature Table; **www.ncbi.nlm.nih.gov/collab/FT/index.html**.
4. GAME XML documentation; **www.fruitfly.org/annot/game-xml.dtd.txt**.
5. Generic Model Organism Database (GMOD) homepage; **www.gmod.org**.
6. GFF2 documentation; **www.sanger.ac.uk/Software/formats/GFF/GFF_Spec.shtml**.
7. GFF3 documentation; **song.sourceforge.net/gff3.shtml**.
8. Kulikova T, Aldebert P, Althorpe A, *et al*. 2004. The EMBL Nucleotide Sequence Database. *Nucleic Acids Res* **32**: 27–30.
9. Lewis SE, Searle SMJ, Harris N, *et al*. 2002. Apollo: a sequence annotation editor. *Genome Biology* **3**: research0082.
10. Miyazaki S, Sugawara H, Ikeo K, Gojobori T, Tateno Y. 2004. DDBJ in the stream of various biological data. *Nucleic Acids Res* **32**: 31–34.
11. Open Biological Ontologies (OBO); **obo.sourceforge.net**.
12. Rutherford K, Parkhill J, Crook J, *et al*. 2000. Artemis: sequence visualization and annotation. *Bioinformatics* **16**: 944–945.
13. Sequence Ontology homepage: **song.sourceforge.net**.
14. Stein LD, Mungall C, Shu S, *et al*. 2002. The generic genome browser: a building block for a model organism system database. *Genome Res* **12**: 1599–1610.

Copyright © 2005 John Wiley & Sons, Ltd.
